# Staying in a punishing place: online narratives about pregnancy and abortion in pre-liberalisation Ireland

**DOI:** 10.1080/26410397.2025.2481761

**Published:** 2025-04-14

**Authors:** Niamh Skelly

**Affiliations:** Lecturer in Mental Health, Division of Mental Health, Midwifery and Health, University of the West of Scotland, Paisley, UK.

**Keywords:** abortion, fetal abnormality, Ireland, miscarriage, mental health, stillbirth

## Abstract

Restricted abortion access impinges on the human rights and health of a significant number of women globally. The reproductive justice framework, as well as recent calls for the normalisation of abortion, encourage examination of the deleterious effects of abortion restrictions. This study explores the self-generated, online narratives of women who experienced crises in pregnancy while living in a restrictive context, namely pre-2019 Ireland, and who did not travel for abortion care. Mental health and emotional experiences are a specific focus. From an archived version of posts to the *In her Shoes – Women of the Eighth* Facebook page made in 2018–2019 (*N* = 728), 96 personal narratives were sampled. Narratives that did not feature travel for abortion care (*n* = 25) were selected for thematic analysis, which was completed by a single researcher in 2024. Themes that emerged included waiting for intervention as a form of mental torture, fear during self-managed abortion, attempts to self-induce abortion driven by despair, and variation in the extent to which proceeding with the pregnancy was a choice. Most women who stayed in place had been constrained by circumstances in deciding to do so. These results enrich our understanding of the negative effects of restrictive contexts on women’s emotional wellbeing. They also draw attention to those who are effectively trapped in restricted contexts and overlooked when the literature narrowly focuses on outward travel from restrictive contexts for abortion care.

## Plain language summary

Being able to access abortion is an important human right. It is also important for women’s health. Abortion was not available in Ireland until 2019. The *In Her Shoes* Facebook page posted the stories of women living in Ireland who became pregnant before 2019. This study examines 25 stories posted on this Facebook page. The writers of these stories did not leave Ireland to have an abortion. Some knew that their babies were unwell and would not survive. They described being forced to wait for medical care as emotionally very difficult. Others ordered abortion pills and took them at home. They described being very frightened during this experience. A small number tried to cause a miscarriage. They did this by hurting their bodies in many ways. Several women continued their pregnancies. Some described this as a choice. Others described feeling forced. Overall, this study demonstrates that not having access to abortion can harm women. One way it can harm women is by causing mental and emotional suffering. Not all women can travel to access abortion. It is important to study the experiences of those who travel, but also those who do not. Both groups face emotional challenges.

## Résumé

Un accès restreint à l’avortement porte atteinte aux droits humains et la santé d’un nombre important de femmes dans le monde. Le cadre de justice reproductive, ainsi que les récents appels à la normalisation de l’avortement, encouragent l’examen des effets délétères des restrictions à l’avortement. Cette étude explore les récits publiés en ligne par des femmes qui ont connu des crises pendant la grossesse alors qu’elles vivaient dans un environnement restrictif, à savoir l’Irlande d’avant 2019, et qui n’ont pas voyagé pour se faire avorter. La santé mentale et les expériences psychologiques en constituent l’axe principal. À partir d’une version archivée de publications de 2018–2019 sur la page Facebook « In Her Shoes – Women of the Eighth » (*n* = 728), 96 récits personnels ont été échantillonnés. Les récits qui ne faisaient pas état de déplacement pour soins d’avortement (*n* = 25) ont été sélectionnés pour l’analyse thématique, réalisée par un seul chercheur en 2024. Parmi les thèmes abordés figuraient l’attente de l’intervention perçue comme une forme de torture psychologique, la peur pendant l’avortement autogéré, les tentatives pour provoquer une interruption de grossesse motivées par le désespoir et les variations dans la mesure où la poursuite de la grossesse était un choix. La plupart des femmes restées sur place avaient été contraintes par les circonstances à faire ce choix. Ces résultats enrichissent la compréhension des effets négatifs des contextes restrictifs sur le bien-être psychologique des femmes. Ils attirent aussi l’attention sur celles qui sont effectivement piégées dans des contextes restrictifs et négligées quand les publications se concentrent étroitement sur les déplacement hors de contextes restrictifs pour obtenir des soins d’avortement.

## Resumen

El acceso restringido al aborto vulnera los derechos humanos y la salud de un considerable número de mujeres a nivel mundial. El marco de justicia reproductiva, así como los llamados recientes a la normalización del aborto, invitan a examinar los efectos perjudiciales de las restricciones al aborto. Este estudio explora las narrativas autogeneradas en línea de las mujeres que sufrieron crisis durante el embarazo mientras vivían en un contexto restrictivo, es decir, en Irlanda antes de 2019, y que no viajaron en busca de servicios de aborto. La salud mental y las experiencias emocionales son un enfoque específico. Desde una versión archivada de *posts* en 2018–19 hasta la página en Facebook titulada *In Her Shoes – Women of the Eighth* (*N* = 728), 96 narrativas personales formaron parte de la muestra. Las narrativas que no implicaban viajar para recibir servicios de aborto (*n* = 25) fueron seleccionadas para el análisis temático, que fue realizado por un solo investigador en 2024. Los temas emergentes fueron: esperar una intervención como forma de tortura mental, temor durante la autogestión del aborto, intentos de autoinducir un aborto impulsados por la desesperación, y variación en el grado en que continuar con el embarazo era una opción. La mayoría de las mujeres que permanecieron en su lugar se vieron limitadas por las circunstancias al decidir no viajar. Estos resultados enriquecen la comprensión de los efectos negativos de los contextos restrictivos en el bienestar emocional de las mujeres. También dirigen la atención a aquéllas que efectivamente están atrapadas en contextos restringidos y son olvidadas cuando la literatura se enfoca estrechamente en viajar fuera del contexto restrictivo en busca de servicios de aborto.

The United Nations (UN) recognises access to safe abortion as a human right.^1^ Comprehensive abortion access constitutes essential healthcare according to the World Health Organization (WHO).^2^ However, the WHO Global Abortion Policies Database (2024) indicates that only 51 member countries permit abortion upon a woman’s request. Of the 195 UN member countries, 81 have abortion laws that are broadly supportive of sexual and reproductive health rights, but 37% of these countries also place unsupportive restrictions on abortion access.^3^ Globally, 22% of women of reproductive age live in countries where abortion is permitted only to save the life of the woman and 6% in countries where it is not permitted under any circumstances.^4^ Despite a general trend towards the liberalisation of abortion law, some countries are increasing restrictions, mostly notably the United States of America. Abortion is now effectively banned in 13 American states, with a further four states imposing a six-week gestational limit.^5^

Until 2019, Ireland’s abortion laws were among the most restrictive in the world.^6^ The eighth amendment to the constitution, which recognised the pregnant woman and unborn child as having an equal right to life, functioned as a constitutional ban on abortion in almost all circumstances.^7^ The eighth amendment was repealed by referendum in May 2018,^8^ allowing for the introduction in January 2019 of a system of abortion upon request up to 12 weeks’ gestation.^9^

The mental health impact of living in a context that restricts legal access to abortion is under-studied. Until relatively recently, empirical studies regarding abortion and mental health were predominately framed in terms of the risks posed by abortion.^10^ Questioning this framing, in keeping with the recent theoretical currents of de-problematising, de-troubling and normalising abortion,^11–13^ highlights alternative avenues for exploration: in what ways are women imperilled by the denial of reproductive agency? How is their mental health negatively affected by living in a restrictive context? A recent longitudinal study examined the association between legal restrictions on abortion access and suicide rates in women of reproductive age, finding a positive relationship.^14^ McKetta et al^15^ found restrictive abortion legislation to be associated with more unintended pregnancies, which were in turn associated with higher levels of pre-natal stress and depression.

The reproductive justice framework focuses on reproductive justice for all and in every sense*.* It is an inherently social and intersectional framework and considers how factors such as marginalisation, racialisation and poverty increase vulnerability to the denial of reproductive agency.^16^ It also contextualises abortion access as one facet of reproductive agency.^17^ It critiques the language of choice employed in some activism, arguing that freedom of choice is differentially distributed according to intersections of identity.^18^ The reproductive justice framework thus compels consideration of who is the most vulnerable to the deleterious effects of restrictive contexts.

Several studies have focused on women’s experiences of clandestine medical abortions in countries with heavily restricted access.^19–22^ There is a growing literature on experiences accessing abortion, including at-home medical abortion, among those living in American states with limited or no provision.^19,23^ There is also an American literature on experiences of travelling to obtain care out of state,^24,25^ some of which consider the emotional impact of such travel.^26,27^ Travel by Irish women to receive abortion care has received considerable academic (See Gilmartin and White^28^) and media (e.g. Baron^29^, and Mackey^30^) attention relative to abortion travel in other regions and by other groups.^31^ However, not until shortly before the 2018 referendum did studies start to appear in the literature that examined the impact of Ireland’s abortion law from the perspectives of women living in Ireland.^32–34^

Women with abortion histories who live in restrictive contexts can be a hard-to-research population. Several studies have utilised online, self-generated pregnancy and abortion narratives, often shared for activist or political purposes, as data.^35–39^ Studies from various disciplines have analysed publicly available narratives shared online during the Irish referendum campaign,^34,40,41^ with a predominant focus on the experience of travel to obtain abortion care. As argued by Mishler,^41^ this focus on travel risks obscuring the experiences of those who do not travel. Crucially, from a reproductive justice perspective, it also precludes grappling with the structural factors that hold many women in place.

## The current study

The current study aims to understand the experiences of women who face crises in pregnancy while living in a jurisdiction that heavily restricts abortion access, specifically pre-2019 Ireland. Such crises include unplanned pregnancies, diagnoses of a significant or fatal fetal abnormality, and miscarriages which require medical intervention. It adopts a qualitative approach, utilising data that were collected via the *In Her Shoes – Women of the Eighth* project and shared publicly and anonymously online.

This study focuses on the experiences of those who remained in Ireland (*stayed in place*), rather than travelling to access abortion services. A reproductive justice lens, which emphasises inequity, influenced this choice. This lens generates curiosity about those who are not mobile in the context of abortion, the possible barriers to their mobility, and the emotional impact of remaining in a restrictive context. This choice was also influenced by a growing emphasis in abortion activism on abortion pills by post as a pragmatic option in restrictive contexts. The question arises whether the shame and fear evident in travel narratives^34,40^ is alleviated when pills can be used to have an abortion at home, or whether the clandestinity of the method gives rise to an emotional experience that shares similarities with that of travel.

The mental and emotional health of women is a focus of this study. Specifically, in contrast to how questions regarding mental health and abortion are usually framed,^10^ the study focuses on the emotional impact of not having access to abortion. It takes a much broader view than psychiatric risk or diagnostic categories. In an Irish context, media and legal discourse for decades prior to the 2018 referendum focused on suicidality, and specifically on whether suicidality in a pregnant women could be grounds for, and resolved by, abortion.^42^ This study seeks to broaden the scope of discussion and consider the many ways that abortion and emotional well-being are potentially intertwined in women’s lived experiences.

## Method

A secondary, qualitative analysis was undertaken of narratives shared online as part of the *In Her Shoes – Women of the Eighth*^43^ project. This project was launched on Facebook and Twitter in January 2018. It was positioned as pro-repeal of the eighth amendment to the Irish constitution. Individuals submitted anonymous narratives detailing “what life looks like when abortion is illegal”.^44^ The majority of the narratives were women’s first-person accounts of pregnancy. They were published prior to and for several months following the May 2018 referendum on the eighth amendment.

Data were accessed via the Digital Repository of Ireland’s (DRI) *In Her Shoes* archive, to which the author was granted access for research purposes. As detailed by Grimes, Cassidy, et al,^45^ the archiving process involved further anonymisation of the narratives. DRI’s *Notice and Action* policy for the archive states that a narrative will be removed if DRI are contacted by the author and they indicate that they do not consent to archiving. Ethical approval for the current study was granted by the University of the West of Scotland’s School of Health and Life Sciences Academic Integrity and Ethics Committee (submission reference 17545) in March 2024.

### Sampling

The DRI *In Her Shoes* archive consists of 958 items, each representing a post on the project Facebook page. Of these, 728 are personal narratives. Using a random number generator (https://www.calculator.net/random-number-generator.html), 96 items from the archive (10%) were initially sampled. A dataset was created based on an item meeting all the following criteria:

1. Item represents a personal narrative about pregnancy/pregnancies and contains sufficient information about how pregnancy ended or is likely to have ended.
2. Person was living in either (the Republic of) Ireland or Northern Ireland at time of the pregnancy.
3. Narrative refers to a pregnancy that occurred prior to the expansion of access to legal abortion.

An item that did not meet these criteria was replaced with the next item from the archive that met all criteria. The dataset for the current study was further refined by application of a fourth criterion:

(4) Person did not travel outside of the island of Ireland during the pregnancy to receive abortion care (i.e. stayed in place).

Items that did not meet the fourth criterion were retained for a separate analysis.

Narratives relating to Northern Ireland were included for two reasons. First, abortion law in Northern Ireland was extremely restrictive until October 2019.^46^ Generally, Northen Irish women had to travel to another United Kingdom nation to access abortion care. Thus, women on both sides of the Irish border were living in similar legal and geographical contexts. Second, Northern Irish women submitted narratives to *In Her Shoes*, and it is possible that some narratives relate to Northern Ireland without explicitly stating this. Additionally, for the purposes of this study, travel to Northern Ireland to collect abortion pills was considered staying in place.* This decision was made after a preliminary review of the larger *n* = 96 dataset, which indicated that the collection of pills in the North and return to the Republic were discussed in different terms to travelling to another part of the United Kingdom and remaining there while receiving abortion care. The latter (which involves travel by sea or air) was framed as travel. The former (which involves crossing an open land border) was framed as a precursor to the process of having an abortion at home.

### Analysis

Sampled narratives were entered into NVivo 12 Pro. Coding was inductive and followed the processes of thematic analysis.^51^ The results and discussion sections of other qualitative studies of women’s abortion experiences were not read in advance of analysis, although other, generally relevant papers were read in full. Adopting a social constructivist framework, the author wanted to root the analysis initially in the language of the writers and to ensure openness to the varying styles and foci of the narratives. Special care was given to familiarisation with the data, as there was no transcription process during which immersion could occur. It was following familiarisation with, and initial, open coding of the sampled narratives (*n* = 96) that the decision was made to focus on the experiences of those who stayed in place. These narratives were organised into groups based on interventions the women underwent and/or their pregnancy outcomes. Narratives were then re-read, compared to the other narratives within the same group, and coding refined. Compared to the open and general initial coding, subsequent rounds were focused on emotion, mental health, and aspects of experience that appeared salient to these. This was a general focus, however, and the use of a pre-designed or rigid coding framework was avoided. Writing was central to the analysis process and assisted both the development of within-group themes and in the analysis of patterns extending across the groups. A reflective diary was kept throughout the analysis process. A draft analysis was presented to a group of research colleagues, for sense-checking of content as well as consideration of quality and rigour. Feedback from this group was incorporated into the final analysis.

### Reflexivity

The author is a female clinical psychologist with an interest in the intersection of physical and mental health. The author grew up in Ireland, which involved exposure to (and normalisation of) orthodox Catholic perspectives on abortion. It also involved exposure to the harms experienced by women due to the inaccessibility of abortion and the deep-seated stigma attached to both abortion and having a child outside of marriage. The author read in real time, prior to the 2018 referendum, many of the narratives published by the *In Her Shoes* project. Being an Irish woman, and living through this change in the rights afforded to women in Ireland, drives the author’s interest in reproductive justice, and desire to analyse and understand the consequences of reproductive injustice.

## Results

Included narratives (*n* = 25) are summarised in Table 1. It was not judged necessary to sample further staying in place narratives from the archive beyond these 25. The aim was not to exhaustively catalogue all possible experiences but rather to provide a sufficiently deep and rich analysis of a random selection of experiences represented in the archive. It was judged that depth and richness would be sacrificed if the study dataset were expanded further.

**Table 1.** Characteristics of included narratives

Narrative	Group	Writer	Age at time of pregnancy	Children already	Year	Fetal diagnosis	Requested or desired intervention	Outcome	Note
1	Miscarriage/non-viable/fetal abnormality	Self	19	No	∼2010	N/A	Surgical management of miscarriage (delayed)	Had surgery; hospitalised for infection post-surgery	
2		Self	DK	Yes – 1	∼2010	Edward’s Syndrome	Induction at 31 weeks (denied)	Stillbirth at 39 weeks	
3		Self	DK	No	DK	N/A	Abortion of non-viable pregnancy (denied)	Miscarried at home	
4		Student nurse	DK	DK	2007		Pre-term induction (denied)	Stillbirth in hospital bathroom	
5		Self	DK	Yes – 1	2017	N/A	Abortion of ectopic pregnancy (delayed)	Abortion of ectopic pregnancy	
6		Self	20	No	∼2010	Perinatal stroke and hydrocephalus	Abortion (denied)	Induced at 36 weeks. Either stillbirth or baby died shortly after birth	
7		Self	DK	Yes – 1	DK	Sirenomelia	Abortion in context of repeated heavy bleeding, oligohydramnios, and FFA (denied)	Induced when no fetal heartbeat detected. Stillbirth	
8		Self	DK	Yes – 1	2018	Yes – not specified	Abortion (denied)	Induced at 24 weeks. Stillbirth	
9		Self	DK	No	∼2017	Down Syndrome and related fatal complications		Stillbirth	IVF pregnancy
									
10	Attempted self-inducement	Self	19	No	2008	N/A		Likely miscarried (did not take pregnancy test)	Lived in Northern Ireland. Tried to self-induce abortion
11		Self	DK	No	∼2008	N/A		Ambiguous (did not take pregnancy test). Miscarried or was not pregnant	Raped. Tried to self-induce abortion
12		Self	17	No	DK	N/A		Miscarried	Tried to self-induce abortion
13	Ordered abortion pills	Self	27	Yes – 1	DK	N/A		Ordered pills online. Had at-home abortion	
14		Self	19	No	DK	N/A		Ordered pills online. Had at-home abortion	
15		Self	DK	Yes – 1	DK	N/A		Ordered pills online. Attempted at-home abortion	Incomplete abortion. Subsequently travelled to UK for surgical abortion
16		Self	19	No	∼2014	N/A		Ordered pills online. Had at-home abortion	
17		Self	DK	Yes – 4	DK	N/A		Ordered pills online. Had at-home abortion	
18^a^		Self	20	No	2017	N/A		Ordered pills online. Had at-home abortion	Tried to self-induce abortion
19	Proceeded with pregnancy	Self	27	No	∼2017	N/A		Gave birth, raising child	Raped
20		Self	19	No	Early 1990s	No		Gave birth, raising child	Concealed pregnancy
21		Self	21	No		No		Gave birth, raising child	Raped
22		Self	23		∼2017	No		Gave birth, raising child	
23		Child	DK	Yes	Early 1980s	No		Gave birth, raised child	Stroke survivor
24		Self	32		DK	No		Gave birth, raising child	
25		Self	DK		DK	Yes – not specified		Gave birth, raising child	

Note: N/A means pregnancy ended before routine screens for fetal abnormalities undertaken; FFA = fatal fetal abnormality; ∼ indicates year is approximate.

^a^ Also included in group that attempted self-inducement of abortion.

Within these 25 narratives, four minimally overlapping subgroups† were identified: women experiencing miscarriages/non-viable pregnancies or who received a diagnosis of a serious fetal abnormality (*n* = 9); women who tried to self-induce abortion (*n* = 4); women who ordered abortion pills online and had at-home, unsupervised medical abortions (*n* = 6); and women who continued with their pregnancies, gave birth and were raising the child (*n* = 7). Specific emotional experiences were salient in each of these groups, and these experiences (of “mental torture”; despair; fear, and a continuum of choice and distress, respectively) are first discussed in turn. Patterns across the groups are then considered, specifically the authors’ relationships to travel and to the place in which they remained.

A number in parentheses after an extract denotes the narrative from which the data are taken (see Table 1). “ … [] … ” indicates that narrative content has been omitted. Information in square parentheses indicates that it was added to the narrative for clarity by the study’s author.

### The torture of forced waiting for intervention

Lack of agency over their pregnancies was prominent in the narratives of those who experienced miscarriage, non-viable pregnancies and serious fetal abnormalities (*n* = 9). This was in contrast to their experiences of becoming pregnant; this was the only group in which some authors (*n* = 5) identified their pregnancies as planned. Many in this group experienced periods of medical inaction, during which they requested but were denied intervention. One woman with a previous fallopian rupture described how she exercised agency; she was “very proactive” in seeking medical care when she experienced pain and bleeding. However, in hospital she felt her wishes were ignored:

*“The hospital made me wait for 1 week before they agreed to terminate the pregnancy. They said they had to be 100% sure it was ectopic. Even with my medical history and the scan showing no pregnancy the[y] made me wait for 5 hcg blood readings* … *[]* … *I was in so much pain they had to give me pethidine, yet they still made me wait.”* (5)

This lack of agency over her pregnancy was specifically stated by hospital staff when she was told she “had no right to” demand earlier intervention. Others in this group also described how their agency was negated by the inaction of medical professionals. However, medical professionals were also sometimes framed as powerless to act:

*“I was told to wait and let things unfold naturally* … *[]* … *all I wanted was for the whole thing to end. I called them again and said I couldn’t wait. I was told there was nothing that they could do.”* (3)

*“We were told there was no option to terminate* … *[]* … *We were forced to continue with the pregnancy* … *[]* … *My waters broke at 34 weeks and I was kept in hospital for two weeks until they were allowed to induce me.”* (6)

*“We were not allowed [an induction] at that time and we would have to wait until I was full term. It was a ‘process’ that we had to go through.”* (2)

Women in this group described believing that their health and lives were in danger, but that nothing was being done, resulting in fear and a sense of neglect:

*“Every day was utterly terrifying. I was afraid to sleep in case I would bleed out in the night, leaving behind my husband and child without a wife or mother* … *I couldn’t be left on my own in case I haemorrhaged again and I was already weak and depressed* … *[]* … *I felt invalidated, uncared for by my country.”* (7)

*“Not only did I want to save my tube but I didn’t want to die. I sat in that hospital worrying about who will take care of my son if I die.”* (5)

In addition to concerns about her own health, the writer of narrative 7 was concerned about her baby’s suffering: *“I worried about the lack of fluid around my baby and [the baby] being crushed”*.

Several narratives described enforced waits that had concrete negative consequences. The writer of narrative 2 *“clung to the hope that our son would live through his birth so we could look into his eyes”* but by the time induction was permitted *“there was no heartbeat. We were too late”.* The writer of narrative 4 was a student nurse who found *“a woman on the toilet floor and her stillbirth baby in the toilet bowl. The woman was quietly crying and the hospital bathroom tiles were covered in blood”.* The woman was waiting for an induction, which was not permitted until no fetal heartbeat was detected. Finally, the writer of narrative 5 described being told by doctors that her ectopic pregnancy had “more than likely” damaged her “only remaining tube”. She expressed her strong desire for another child but stated that because of the forced wait for treatment *“I may never be able to have any more children”*.

As reflected in some of the above extracts, women described significant distress during these waiting periods. Some also mentioned the negative effects on close family members. Four writers referred to their experiences as “torture”:

*“I had 2 mental breakdowns, one happened on my birthday and I was hospitalised for 4 days* … *[]* … *it was decided that I needed to be medicated. I was unable to look after my son.”* (2)

*“I spent the next five days crawling the walls, in an almost catatonic state. It was horrendous. It felt like torture. It was torture.”* (3)

*“The hospital visits and scans were torture. Witnessing women on the ward going into labour was torture* … *[]* … *They were 5 of the hardest months of my life.”* (6)

In addition to the distress they experienced while waiting, the women in this group also described ongoing negative effects on their mental health:

*“I will never get over this, the extended and unnecessary trauma of this.”* (7)

*“This still makes me cry so hard. I cannot ever get over how I was left to just deal with it.”* (3)

*“I am still in a form of hell but one that gets easier to live in as each day goes by.”* (8)

### The despair of trying to self-induce

In two narratives regarding attempted self-inducement of an abortion, intense despair was apparent. Both writers described sustained engagement in a wide variety of self-injurious behaviours. Seeking to induce an abortion involved significant physical suffering for both. The writer of narrative 12 *“ended up in hospital with stomach ulcers and dehydration”.* The writer of narrative 11, who was fearful of pregnancy following rape, was so despairing that if unable to avoid pregnancy through self-injury, she was decided on suicide:

*“I* … *[]* … *bought in bulk any type of painkiller I could, along with prescription painkillers and steroids that had previously been prescribed* … *[]* … *They were my back up* … *[]* … *If I was pregnant I would use those pills to kill myself.”*

The writer of narrative 10 engaged in less extreme self-injury, but nonetheless over several days punched her stomach so hard *“there were red welts and slap marks for a long time”.* She had an upcoming, pre-arranged trip to a Scottish city, but did not have the money to pay for abortion care. However, she expressed less despair; rather than the alternative being suicide, she wrote that obtaining an abortion in Scotland “*could have been my story”* had she remained pregnant. For the writer of narrative 18, it was her initial fear regarding accessing abortion pills that led her to engage in risky, potentially self-injurious behaviours:

*“I started going through my medicine cabinet, finding random drugs and taking them* … *[]* … *I started drinking heavily and picked up smoking in hopes that maybe that would cause some kind of miscarriage.”*

These behaviours did not result in the pregnancy ending, and despite financial constraints she later purchased abortion pills online.

### Fear in at-home medical abortions

Fear was the dominant emotion in narratives that described ordering abortion pills online and taking them without medical supervision. Reasons for this fear varied:

*“I am so scared to take them [the pills] as I will not be supervised.”* (13)

*“I took the abortion pill and within an hour I started the most terrifying experience of my life* … *[]* … *I thought I was going to die.”* (18)

*“I took them at home* … *[]* … *constantly worrying that something would go wrong and I’d be rushed to hospital not knowing what to say* … *[]* … *I was so worried about getting an infection or something and there was nowhere I could go for a follow-up.”* (17)

Several women mentioned experiencing intense fear in relation to finding themselves unintentionally pregnant. One experienced this fear as more intense than her fear of an unsupervised abortion:

*“I wasn’t brave, I was too desperate and too scared of the future to be afraid of what was happening.”* (16)

For one woman, fear and lack of legal abortion access played a complex role in her decision to have an unsupervised medical abortion. Her unplanned pregnancy represented the realisation of her “biggest fear”, despite very much wanting to have another child. She feared experiencing complications during pregnancy related to an underlying medical condition, and being refused treatment:

*“My life needs to be in imminent danger before they can act, and what if that’s too late? What if they are unable to save me because they missed the ‘window of opportunity’ due to the 8th?”* (13)

The restrictive legal context presented too much risk, and generated too much fear, for her to proceed with the pregnancy:

*“I am not having an abortion because it is what I want* … *[]* … *I am too scared to risk my life to create another.”* (13)

Regarding the overall emotional impact of these experiences, the writer of narrative 13 concluded by describing herself as “broken”. In narrative 18, another woman described how she *“spiralled into a depression and took to smoking weed to try and numb the pain”* after her abortion. The writer of narrative 16 described lingering anger and hurt regarding the risks she had to take to have an abortion, and the isolation it engendered, as well as “profound sadness” for her younger self. For another, the need for time to recover from an emotionally difficult experience sat alongside confidence in her decision:

*“A year later I’m finally getting over the stress of it all (barely)* … *[]* … *I KNOW I made the right decision for my family and I don’t regret it at all.”* (17)

The two counterexamples to these fear-dominated narratives highlight how having access to information regarding at-home abortion can potentially transform the emotional experience. One of these counterexamples is provided by a woman who ultimately proceeded with her pregnancy:

*“I knew my options* … *[]* … *I knew which websites to visit to get pills sent to me* … *[]* … *I knew if things went wrong I could go to the hospital and just tell them I was having a miscarriage – they couldn’t tell the difference. I wasn’t afraid of getting caught or going to prison.”* (24)

The writer of narrative 14, with the support of a friend, accessed helpful online information and *“was very comfortable on completing the medical abortion at home”.* Moreover, when she experienced a serious post-abortion complication, she attended hospital and made no reference to fear as a barrier to doing so.

### Degrees of choice in proceeding with pregnancy

There was a continuum of those who proceeded with their pregnancy (*n* = 7). Some felt constrained by circumstances. Following a diagnosis of fetal abnormalities, the writer of narrative 25 was not given practical information about options (*“we were* … *[]* … *only told to google what we needed”*) and did not receive emotional support such that she could make an informed decision (*“I was so angry and out of my mind I couldn’t process the information”*)*.* Distress about potential judgement also influenced her decision to continue with the pregnancy:

*“The thoughts of travelling and everyone knowing the shame that I had given up on our son left me lying awake at night. Instead we ignored advice and blindly proceeded with the pregnancy. Our son was born with severe disabilities.”* (25)

The writer of narrative 20 had a concealed pregnancy. Travelling for abortion care was impossible, and she described how intense emotions and a hostile socio-cultural context also influenced her decision to proceed, but in secret.

The writer of narrative 19 became pregnant following rape. Similar to narrative 20, she felt she had no options regarding the pregnancy, as she lacked the financial means to access abortion abroad. Another woman who proceeded with her pregnancy was also raped, but did not know if her pregnancy was a result of the rape. She made several attempts to arrange travel to England for abortion. Fear of travelling alone post-abortion and financial constraint were factors in her decision making. From her descriptions, her decision to proceed was not freely made:

*“I’ve never felt so trapped in my entire life* … *[]* … *I felt like my choice had been taken away from me* … *[]* … *I thought and thought and agonized over this decision. Ultimately, I decided to keep the baby.”* (19)

Two narratives describe more freely made decisions. In both cases, the women felt they had the necessary information regarding options, and both implied they had the means to access abortion. Through involvement in pro-choice activism, one had received training regarding accessing and utilising abortion pills. She expressed confidence that this was a safe, accessible option for her, but ultimately not one she wished to avail of. Although she described pressures relating to housing, work and lone parenting, she proceeded because of her desire to be a mother. The other author who described proceeding with her pregnancy in positive terms described a supportive partner. She also described benefitting from accessing information and support via a pro-choice counselling service:

*“we made an appointment with a crisis pregnancy counsellor in [names specific service]* … *[]* … *talked through our options in a non-judgemental, factual way. Talking it through with someone impartial definitely helped and I left the centre much calmer and with a clearer head.”*

The women who proceeded with their pregnancies in constrained circumstances described periods of distress and difficulty in parenting:

*“It was impossibly hard so hard at times that I thought my life wasn’t worth living.”* (20)

*“Thankfully I had amazing family and friends who stepped up and basically raised my daughter for those first few months when I locked myself away.”* (21)

For the author of narrative 25, whose child was diagnosed with serious health difficulties pre-natally, this distress was related to her child’s physical symptoms and the guilt she experienced regarding his quality of life:

*“But when people say I wouldn’t change a thing. I would. In a heartbeat. When I see him struggle, drowning in his own body I feel so much guilt for* … *[]* … *putting him through this torture.”*

These experiences contrasted with the descriptions of mothering provided by the two women whose decisions to proceed with their pregnancies were more freely made. Their narratives did not contain the same intensity of distress, focusing instead on how proceeding positively impacted their lives. However, both emphasised the importance of proceeding being a choice.

*“My heart burst with pride the moment I met her and I can’t describe how much love I feel for this tiny little girl, she’s my world* … *[]* … *To continue with my pregnancy was the right choice for ME, but that doesn’t mean it’s the right choice for every woman!”* (22)

*“My child is my joy, my delight. The centre of my world. How dare anyone call my child a punishment?* … *[]* … *I am still passionately pro-choice. More so.”* (24)

### Staying in a punishing place

Across all 25 narratives, nine made no mention of travel for abortion care or stated only that it was not possible. Reasons for not travelling may be inferred from some narratives, such as that physical or mental health posed a barrier:

*“I couldn’t be left on my own in case I haemorrhaged again and I was already weak and depressed.”* (7)

*“I sat in the hospital every minute of every day waiting for my only remaining tube to rupture.”* (5)

For the woman described in narrative 23, travel would not have provided a ready solution; her stroke-related care was unilaterally “*put on standby*” by her doctor because of her unplanned pregnancy.

There is an indirect reference to travel in narrative 2, which highlights the double bind of those who received fatal fetal abnormality diagnoses:

*“We got to say goodbye to our son in our own country. We left the hospital, with our son’s tiny white coffin between us in the back of the funeral car. He had a lovely funeral. We were surrounded by our loving family at all times. Was it worth waiting 8 weeks, my answer is No.”*

Those who wished to end their pregnancy either had to travel and endure one form of suffering, or wait and endure another. Both forms of suffering were avoidable, generated by the restrictive legal context (and imposed on families already experiencing the grief of their babies’ diagnoses). Relatedly, the writer of narrative 9 judged the suffering involved in travel as more than she could bear (*“I would never have survived a journey to or from England”*). She instead prayed that her baby would die *in utero*, to spare the baby the “*agonising pain*” predicted by doctors if born alive.

The most frequently stated barrier to travel, appearing in nine narratives, was financial constraint, sometimes combined with other factors:

*“I couldn’t afford to go to the doctor let alone travel to England for an abortion.”* (12)

*“There was no way I could afford to travel for a termination so I looked into buying the pills online.”* (17)

*“We both lived at home and wouldn’t have been allowed away together even if the financial impediment wasn’t there. It sounds ridiculous but that’s how it was.”* (20)

But financial means were not a guarantee of being able to access appropriate care abroad, as described by one woman who was in the second trimester:

*“Shortly after my 20 weeks scan Liverpool hospital could no longer accept Irish patients, a private clinic was not advisable due to my previous labour.”* (8)

Two women stayed because they wanted to have a baby, having considered the alternatives available to them (narratives 22 and 24). Another described abortion pills by post as easier than travel (narrative 14). However, in most narratives, women stayed because they had to. They stayed in the place whose social and legal context they experienced as punishing and uncaring. Some talked about how their suffering was heightened, as they were also grappling with the grief of fatal fetal abnormality diagnoses or struggling to make a decision about their pregnancy. Others identified the legal and social context as the main driver of their distress:

*“It could have been made easier without all the secrecy and stress but not as things are now.”* (17)

*“Why in such devastating circumstances are we punished further, neither are fair.”* (8)

*“I feel angry that I perceived the idea of being pregnant as such a stigma that I couldn’t even ask for help. I am saddened that 17-year-old me was so ashamed and health care was so restrictive that I took such a dangerous path to end my pregnancy.”* (12)

## Discussion

This study is the first to use online narratives to explore the experiences of women living in jurisdictions with virtually no legal abortion access who stay in place when faced with a crisis in pregnancy. It is one of a small number of studies to consider the impact of restrictive abortion laws from the perspective of women living under these laws. Such studies are important given that globally 40% of women of childbearing age live in restrictive contexts.^4^

The findings highlight, first, that highly restrictive abortion law can impact on the care received by, and well-being of, women experiencing miscarriage and ectopic pregnancy. Women experienced delays to or denial of miscarriage care as traumatic, compounding their distress about their pregnancy outcome. The death of Savita Halappanavar in 2012 in an Irish hospital, due to sepsis arising from delayed miscarriage management, highlighted the physical health risks of miscarriage care in abortion-restrictive contexts.^52^ It is important to also recognise the likely widespread mental health implications. The mental and physical health implications of the recent intensification of American abortion restrictions for women experiencing miscarriage is a significant cause for concern.^53^

Second, in keeping with the findings of Aiken, Johnson, et al^32^, these narratives illustrated how the criminalisation of self-management, and the dearth of knowledge regarding self-management that can arise from criminalisation, engenders fear and isolation for some women choosing this route. The restrictive context creates a heightened risk that some women will have a traumatic experience of self-managed abortion. Although no author in this study mentioned availing of telephone or email support regarding abortion pills, the work of Ramos et al^21^ and Baum et al^19^ indicates that availing of such support can reduce distress and lead to a more positive abortion experience. This may also be true in less restrictive contexts, such as Argentina,^54^ where some women continue to prefer or have more ready access to self-managed abortion.

Third, this study aligns with the findings of Aiken, Johnson, et al^32^ and Aiken, Padron, et al^23^ regarding ineffective and sometimes dangerous attempts at self-inducement in restrictive contexts, such as women resorting to excessive alcohol consumption or inflicting physical trauma to their abdomens. Previous research indicates that poverty and youth are risk factors for attempting self-inducement^55,56^; all four authors in the current study who attempted self-inducement appeared to be students and in their late teens to early 20s. The present study also suggests a possible connection between efforts at self-inducement and suicidality, with self-inducement potentially associated with suicidal ideation. The mental health implications of attempting to self-induce require further study, specifically whether such attempts are a risk factor for suicide, especially when abortion is not achieved. It is unclear to what extent the option to buy abortion pills online, even if illegal, reduces attempts at self-inducement, or how such a service needs to be provided to be accessible to those most likely to attempt self-inducement.

Regarding travel, it was notable that most narratives concerning fetal abnormalities and non-viable pregnancies made no mention of travel. Perhaps the barriers to becoming a “sometimes migrant”^34^ to access abortion seemed so evident to these women that it did not feel necessary to state them. These women were in time-sensitive situations, experiencing significant emotional distress and sometimes also physically at risk. Previous studies have discussed the significant physical and emotional toll of travelling to access abortion services, even in the context of a medically uncomplicated pregnancy.^24,32^ This point remains relevant to the post-repeal Irish context. Grimes, Mishtal, et al^57^ evidenced that it is difficult to obtain an abortion in Ireland post-12 weeks, when many fetal abnormalities are diagnosed. Moreover, Chakravarty et al^58^ highlighted that women who remain pregnant after attempted early medical abortions may find themselves beyond the gestational limit of 12 weeks and ineligible for further abortion care in Ireland. Thus there are many women in Ireland who are “still travelling”.^57^ And what of those who are still staying in place? There is a need for further research examining the experiences of women in Ireland seeking abortion post-12 weeks gestation, including those who are denied care and stay in place.

The findings support what has become a pro-choice axiom: that making abortion illegal does not prevent abortions, just safe abortions. However, the findings also illustrate that making abortion illegal *does* prevent some women from having abortions, resulting in significant distress and trauma. These issues are important to analyse from an intersectional perspective. In this likely racially homogenous group of women, it was evident that being young, already being a mother, and being economically disadvantaged impacted on experiences of crises in pregnancy, and constrained choice regarding travelling. The facts of the case of Ms Y‡ illustrate the considerable vulnerability of asylum seeker/international protection applicant women to forced pregnancy and birth in pre-2019 Ireland. Chakravarty et al^64^ and Chakravarty et al^58^ both outlined the continued barriers to abortion access faced by immigrant and racialised women in Ireland post-legalisation. Just as these women are not represented in this sample from the *In Her Shoes* archive, they were not adequately acknowledged and platformed in mainstream pro-repeal activism.^64^ Chakravarty et al^58^ identified specific aspects of current Irish abortion provision that disproportionately impact the most marginalised women, namely mandatory 3-day waiting periods, the requirement of proof of government services registration (in the form of a “PPS number”) and provision for conscientious objectors, which has resulted in abortion care deserts, particularly in less populated areas. Injustices evident pre-repeal, related to class, age, racialisation and immigration status, and partially illustrated in these narratives, are perpetuated post-repeal. Repeal has not been achieved to the same degree for all in Irish society.

A limitation of this study is that the findings do not represent the only or necessarily the dominant experiences of women who confront crises in pregnancy while living in restrictive contexts. However, considered alongside similar previous studies,^23,32^ they likely represent common experiences. A further limitation is that the pregnant women in the sampled narratives are likely a homogenous group in terms of racial identity. This is perhaps unsurprising, given that the Irish population was 87% white in 2022,^65^ and 95% white in 2006,^66^ but means that the study does not directly present the narratives of racialised groups who are particularly vulnerable to reproductive harm in restrictive conducts. Moreover, Irish Travellers, international protection applicants (asylum seekers) and LGBTQ+ individuals do not feature in these narratives, or at least no pregnant individual is identified as belonging to any of these groups. Because the data were collected in an open-ended manner, there is no consistency in terms of establishing each woman’s age or when her pregnancy occurred. There are unknowns that vary from narrative to narrative, and in some cases, there is even slight ambiguity regarding pregnancy outcome (e.g. narrative 6). Finally, the entirety of the analysis of the narratives was undertaken by one researcher, although research colleagues were consulted during the analysis process and feedback incorporated. The equal involvement of an additional researcher in the entire analysis process would potentially have increased study rigour further.

A strength of this study is the use of online abortion narratives. This method has unrealised potential. In restrictive contexts – particularly those where there is evidence of increasing criminalisation of abortion (e.g. Dellinger and Pell^67^) – sampling may be hampered by women’s fear of detection by authorities. It may be particularly difficult for researchers to gain the trust of (or, in some instances, protect) marginalised women, given that they are disproportionately vulnerable to the criminalisation of abortion. Self-generated online narratives circumvent these problems and may allow women to centre the aspects of their pregnancy and abortion experiences that matter most to them (see O' Brien and Clare^68^). As evidenced by the *In Her Shoes* project, they also enable the women most affected by restrictions to drive political change. Online narratives are one way of preventing restrictive contexts from being quiet contexts.

## Conclusion

The policy, advocacy and activist implications of this work are multiple. Above all, women should be provided with holistic reproductive health care that includes access to abortion on request. In all contexts, women who self-manage their abortions via medication should have access to support options such as telephone and email contact. In restrictive contexts, women who experience miscarriages and women who receive fetal abnormality diagnoses are at heightened risk of distress and trauma and may require specialist mental health support. Such women may stay in restrictive contexts not through choice, but because of physical health and mental health barriers to travel, as well as the time-sensitive nature of their need for intervention, making travel impractical. This is in addition to the barriers to travel faced by all pregnant people, such as financial considerations. Mental health support should be available to, and cognisant of the needs of, both those who travel and those who stay in place. These mental health needs are unlikely to be acknowledged and provided for by policymakers who support restricted abortion access, and are thus most likely to be met by third sector organisations. Lastly, policymakers, advocates and activists in all contexts should be alert to the risks of self-inducement and adopt a harm reduction approach. Such an approach would facilitate access to safer means of ending pregnancies that are acceptable to these women (e.g. meet potential requirements for privacy and concealment from close others), such as self-managed medical abortion.

## Supplementary Material

Supplemental Table 1. Digital Repository of Ireland Landing Pages for Included Narratives

## Footnotes

* The land border between Ireland and Northern Ireland is seamless and involves no routine immigration controls.^47^ However, racialised people are more likely to be targeted for checks regarding border crossing by both the Irish police (“Operation Sonnet”; Dáil Deb 5 December 2017 vol 962, no. 6, question 225) and their Northern Irish counterparts (“Operation Gull”^48^). There is thus a “hidden hard border”^49^ for certain marginalised groups. No narratives analysed as part of this study mentioned concerns about crossing the border and racialised identity or immigration status. However, the analysed narratives almost certainly represent only one possible experience of border crossing, namely that of those who are white and have the right to freely travel within the Common Travel Area of Ireland and the United Kingdom. For information on other possible experiences, see Butterly.^50^

† Narrative 18 is counted twice, as a narrative of attempted self-inducement and also as a narrative of abortion via pills received by post.

‡ Ms Y was a teenager who claimed asylum in Ireland and discovered she was pregnant as a result of rape shortly after arrival.^59^ She repeatedly requested an abortion. As her pregnancy progressed, she expressed suicidal intent. There were considerable legal and financial barriers to her travelling abroad for abortion, although she did attempt to do so and was detained by British authorities.^60^ She was denied abortion in Ireland under legislation introduced following the death of Savita Halappanavar.^61^ Her request for an abortion was not formally considered by authorities until the pregnancy had progressed past viability, and due to being past viability she was not eligible for an abortion under the Act.^62^ It was claimed that she consented to a second-trimester c-section, resulting in a live birth, although the circumstances in which she consented to this procedure do not indicate true informed consent.^7^ The child was taken into state care.^63^

## Provenance statement

This article was not commissioned and went through external peer review.
