# Supplementary material for: Staying in a punishing place: online narratives about pregnancy and abortion in pre-liberalisation Ireland
Source: Sex Reprod Health Matters. 2025 Apr 14;33(1):2481761. doi: 10.1080/26410397.2025.2481761 (PMC12086944; doi:10.1080/26410397.2025.2481761)
Supplement: Supplemental Table 1. Digital Repository of Ireland Landing Pages for Included Narratives [file ZRHM_A_2481761_SM2183.docx]

**Additional Materials**

Table 1

*Digital Repository of Ireland Landing Pages for Included Narratives*

| Narrative Number | URL |
| --- | --- |
| 1 | https://repository.dri.ie/catalog/vq28cf58m |
| 2 | https://repository.dri.ie/catalog/3x81mc67p |
| 3 | https://repository.dri.ie/catalog/qb991761x |
| 4 | https://repository.dri.ie/catalog/q237xk09s |
| 5 | https://repository.dri.ie/catalog/s752m558m |
| 6 | https://repository.dri.ie/catalog/cn700x310 |
| 7 | https://repository.dri.ie/catalog/t148v929j |
| 8 | https://repository.dri.ie/catalog/z0302z08m |
| 9 | https://repository.dri.ie/catalog/f188fd073 |
| 10 | https://repository.dri.ie/catalog/np19h335p |
| 11 | https://repository.dri.ie/catalog/0287dp21w |
| 12 | https://repository.dri.ie/catalog/q8120b97t |
| 13 | https://repository.dri.ie/catalog/4q77vj503 |
| 14 | https://repository.dri.ie/catalog/v9807w589 |
| 15 | https://repository.dri.ie/catalog/f762h575h |
| 16 | https://repository.dri.ie/catalog/b564jj71w |
| 17 | https://repository.dri.ie/catalog/st74sh62s |
| 18 | https://repository.dri.ie/catalog/sj13pv290 |
| 19 | https://repository.dri.ie/catalog/8c980h59v |
| 20 | https://repository.dri.ie/catalog/dr27bq54g |
| 21 | https://repository.dri.ie/catalog/dv14cm57j |
| 22 | https://repository.dri.ie/catalog/1z410m17d |
| 23 | https://repository.dri.ie/catalog/td96zv85q |
| 24 | https://repository.dri.ie/catalog/1z410k99p |
| 25 | https://repository.dri.ie/catalog/0574fj871 |

*Note.* Each landing page includes a link to the original Facebook post. These links were still live at the time of preparing this table.
